# Interplay of Digital Proximity App Use and SARS-CoV-2 Vaccine Uptake in Switzerland: Analysis of Two Population-Based Cohort Studies

**DOI:** 10.3389/ijph.2023.1605812

**Published:** 2023-09-20

**Authors:** Paola Daniore, André Moser, Marc Höglinger, Nicole Probst Hensch, Medea Imboden, Thomas Vermes, Dirk Keidel, Murielle Bochud, Natalia Ortega Herrero, Stéphanie Baggio, Patricia Chocano-Bedoya, Nicolas Rodondi, Stefano Tancredi, Cornelia Wagner, Stéphane Cullati, Silvia Stringhini, Semira Gonseth Nusslé, Caroline Veys-Takeuchi, Claire Zuppinger, Erika Harju, Gisela Michel, Irène Frank, Christian R. Kahlert, Emiliano Albanese, Luca Crivelli, Sara Levati, Rebecca Amati, Marco Kaufmann, Marco Geigges, Tala Ballouz, Anja Frei, Jan Fehr, Viktor von Wyl

**Affiliations:** ^1^ Institute for Implementation Science in Health Care, University of Zurich, Zurich, Switzerland; ^2^ Digital Society Initiative, University of Zurich, Zurich, Switzerland; ^3^ Clinical Trials Unit Bern, University of Bern, Bern, Switzerland; ^4^ Winterthur Institute of Health Economics, Zurich University of Applied Sciences, Winterthur, Switzerland; ^5^ Swiss Tropical and Public Health Institute, Allschwil, Switzerland; ^6^ University of Basel, Basel, Switzerland; ^7^ Unisanté, University Center for Primary Care and Public Health, Lausanne, Switzerland; ^8^ Institute of Primary Health Care (BIHAM), University of Bern, Bern, Switzerland; ^9^ Population Health Laboratory (#PopHealthLab), University of Fribourg, Fribourg, Switzerland; ^10^ Department of General Internal Medicine, Inselspital, Bern University Hospital, University of Bern, Bern, Switzerland; ^11^ Department of Readaptation and Geriatrics, University of Geneva, Geneva, Switzerland; ^12^ Department of Health and Community Medicine, Faculty of Medicine, University of Geneva, Geneva, Switzerland; ^13^ Unit of Population Epidemiology, Division of Primary Care Medicine, Geneva University Hospitals, Geneva, Switzerland; ^14^ Faculty of Health Sciences and Medicine, University of Lucerne, Lucerne, Switzerland; ^15^ Clinical Trial Unit, Lucerne Cantonal Hospital, Lucerne, Switzerland; ^16^ School of Health Sciences, ZHAW Zurich University of Applied Sciences, Winterthur, Switzerland; ^17^ Department of Infectious Diseases and Hospital Epidemiology, Cantonal Hospital St. Gallen, St. Gallen, Switzerland; ^18^ Infectious Diseases and Hospital Epidemiology, Children’s Hospital of Eastern Switzerland, St. Gallen, Switzerland; ^19^ Institute of Public Health, Faculty of Biomedical Sciences, Università della Svizzera Italiana, Lugano, Switzerland; ^20^ Department Business Economics, Health and Social Care, University of Applied Sciences and Arts of Southern Switzerland, Manno, Switzerland; ^21^ Epidemiology, Biostatistics and Prevention Institute, University of Zurich, Zurich, Switzerland; ^22^ Division of Infectious Disease and Hospital Epidemiology, University Hospital Zurich, Zurich, Switzerland

**Keywords:** public health, COVID-19, digital proximity tracing, vaccination, public health measures

## Abstract

**Objectives:** Our study aims to evaluate developments in vaccine uptake and digital proximity tracing app use in a localized context of the SARS-CoV-2 pandemic.

**Methods:** We report findings from two population-based longitudinal cohorts in Switzerland from January to December 2021. Failure time analyses and Cox proportional hazards regression models were conducted to assess vaccine uptake and digital proximity tracing app (SwissCovid) uninstalling outcomes.

**Results:** We observed a dichotomy of individuals who did not use the SwissCovid app and did not get vaccinated, and who used the SwissCovid app and got vaccinated during the study period. Increased vaccine uptake was observed with SwissCovid app use (aHR, 1.51; 95% CI: 1.40–1.62 [CI-DFU]; aHR, 1.79; 95% CI: 1.62–1.99 [CSM]) compared to SwissCovid app non-use. Decreased SwissCovid uninstallation risk was observed for participants who got vaccinated (aHR, 0.55; 95% CI: 0.38–0.81 [CI-DFU]; aHR, 0.45; 95% CI: 0.27–0.78 [CSM]) compared to participants who did not get vaccinated.

**Conclusion:** In evolving epidemic contexts, these findings underscore the need for communication strategies as well as flexible digital proximity tracing app adjustments that accommodate different preventive measures and their anticipated interactions.

## Introduction

Digital proximity tracing apps have played an important role in assisting public health measures to mitigate spread of the severe acute respiratory syndrome coronavirus 2 (SARS-CoV-2) in absence of vaccines [1, 2]. Such apps work by anonymously recording the user’s proximity contacts and notifying them in case of a positive SARS-CoV-2 test [3]. As a result, notified individuals can promptly self-isolate, helping to minimize the spread of the virus.

As SARS-CoV-2 vaccines became widely available in 2021, digital proximity tracing apps shifted from a recommended measure in the absence of vaccines to a complementary measure, working alongside vaccination efforts to further minimize the spread of the virus [4, 5]. Such developments raise the question as to whether individuals who previously used digital proximity tracing apps are more likely to seek vaccination for individual or public-level protection. Furthermore, it remains unclear whether individuals become less interested in using digital proximity tracing apps after getting vaccinated [6, 7].

Barriers and facilitators for digital proximity tracing app use, taking preventive measures and vaccine uptake have been discussed extensively in recent papers. Some studies have attributed long-term digital proximity tracing app non-use to a lack of perceived benefits and privacy concerns [8, 9]. Furthermore, studies highlighted the importance of appropriate and fact-driven communication of the vaccines’ benefits to enable their uptake and to encourage continued practicing of preventive measures [10, 11]. Recent evidence also suggests that many individuals exhibit hesitancy towards wearing masks after receiving the vaccine [12], instead opting for alternative, non-invasive preventive measures [13].

However, there is little evidence on the possible effect of SARS-CoV-2 vaccine availability on individual-level decisions to continuously use digital proximity tracing apps. Investigating the interplay between these may offer further insights on the use of novel public health technologies and their possible interactions with other preventive measures. Furthermore, it can provide context on adherence to public health guidance based on different individual-level and public risk-benefit profiles.

### Aims

Our study aimed to explore trends in the uptake of the first SARS-CoV-2 vaccine dose (henceforth referred to as *vaccine uptake*) and the use of Switzerland’s digital proximity tracing app, SwissCovid. We do so by exploring hypotheses based on individual trade-off theories from economics applied to public health [14]. Specifically, we assess if vaccine uptake and SwissCovid app use are motivated by individuals seeking (a) both individual-level protection from vaccine uptake as well as population-level protection through continued use of the SwissCovid app or (b) individual-level protection through vaccine uptake and possible discontinuation of SwissCovid app use after vaccine uptake. We provide context through analyses assessing trends in preventive measures (e.g., maintaining social distance, only leaving the house when necessary) taken by individuals leading up to vaccine uptake or SwissCovid app uninstalling. The goal of this study is to inform communication strategies and digital proximity tracing app development efforts to account for different preventive measures in epidemic or communicable disease contexts.

## Methods

### The SwissCovid Digital Proximity Tracing App

The SwissCovid app was first introduced in Switzerland on 25 June 2020. From its launch until its deactivation on 1 April 2022, the SwissCovid app had around 1.9 million users, which accounted for approximately 26.1% of all Swiss residents aged 16 years and older [15]. Its primary purpose was to detect and isolate possible cases of SARS-CoV-2 infection using an exposure notification cascade system. The SwissCovid app operated in the background, unlike other major apps such as NHS-COVID-19 app in England and the Corona-Warn-App in Germany, which offered additional features such as test result notifications and symptom tracking [16].

### Study Design and Participants

Our study draws on two nationwide longitudinal panel studies conducted in Switzerland throughout the COVID-19 pandemic: (a) the Corona Immunitas Digital Follow-Up eCohort (CI-DFU) study [17] and (b) the COVID-19 Social Monitor (CSM) study [18]. Both studies were designed, in part, to monitor the physical and mental health effects of the SARS-CoV-2 pandemic, adoption of preventive measures and vaccine uptake.

Corona Immunitas is a centrally coordinated research program of population-based seroprevalence studies conducted across Switzerland [17]. Individuals aged 18+ years from the residential registry of participating cantons of the country were randomly selected by the Swiss Federal Statistical Office and invited to participate in the Corona Immunitas study [19]. Participants were then invited to join the CI-DFU, with an exception for two study sites where participants started with the CI-DFU directly [20]. Participants over the age of 65 were oversampled by design in the CI-DFU. As of December 2021, 13,942 participants completed at least one monthly questionnaire in the Basel, Bern, Freiburg, Lucerne, Neuchatel, St. Gallen, Vaud and Zurich study sites. The nationwide Corona Immunitas program ended in December 2021.

The CSM is a population-based online panel survey that started at the end of March 2020. Participants were randomly sampled from the Swiss population aged 18+ years through a Swiss survey company and invited to join the study [21]. Participants who had completed the first survey round were invited to complete follow-up surveys, with a sample replenishment taking place in December 2020 to counteract sample attrition, increasing the study size to 3,381 participants [22].

### Procedures, Outcomes, and Exposures

**FIGURE 1:**
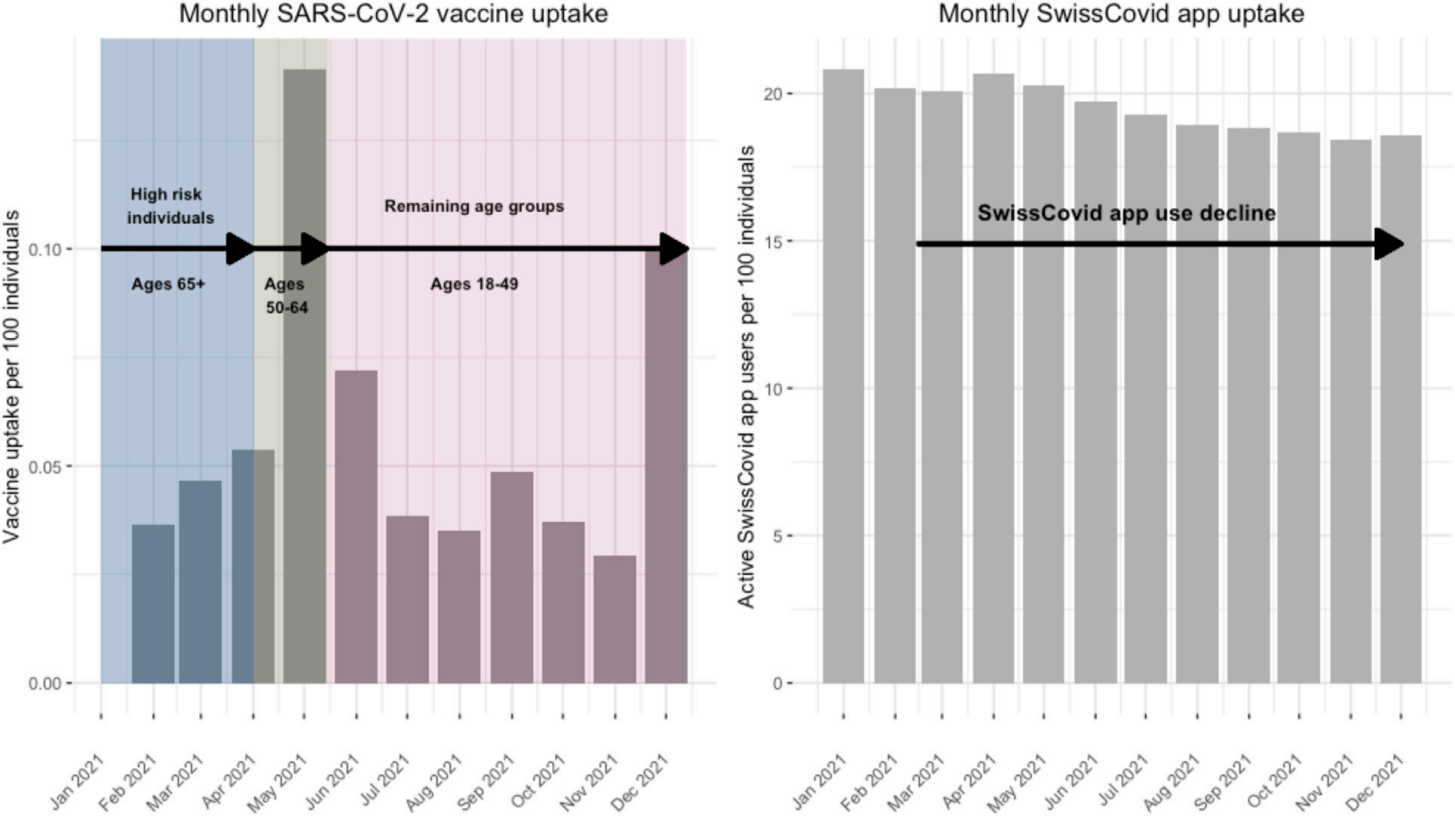
National SwissCovid app use and vaccine uptake data during study period. Switzerland, January to December 2021.

First doses of the SARS-CoV-2 vaccines were made widely available to older adults, populations at risk and healthcare professionals in Switzerland starting January 2021 and were made available to the whole Swiss population from mid-2021 onwards (Figure 1) [23]. In this study, we assessed survey responses between January 2021 and December 2021. We chose this time period because it covered the period from January 2021 when vaccines became available in Switzerland to December 2021 when the nationwide Corona Immunitas program concluded. Additionally, there was an observed decrease in SwissCovid app use between January 2021 and December 2021. For the CI-DFU, we assessed responses from monthly follow-up surveys from 10 January 2021 until 9 December 2021. From the CSM, we assessed responses from 25 January 2021 until 16 December 2021.

We assessed self-reported outcome and exposure measures from the CI-DFU and CSM. The questions in the surveys assessed in our study from both data sources were aligned in terms of content and possible answer choices. As such, the assessments of the outcome and exposure measures of the CI-DFU and CSM were identical. The primary outcomes assessed in two substudies are (1) vaccine uptake and (2) SwissCovid app uninstalling. Participants were assessed until the time to event of interest or right-censoring, depending on which occurred first. The exposures are whether (a) participants actively used the SwissCovid app or (b) got vaccinated during the study period.

Participants were stratified based on when they officially became eligible for SARS-CoV-2 vaccines and allocated to three baseline groups accordingly: (a) ages 65+, (b) ages 50–64 and (c) ages 18–49. Within these groups, we included participants who reported having a severe chronic condition (i.e., cancer, cardiovascular or autoimmune diseases) or who worked as healthcare professionals and received their first vaccine dose before it was made available to their age groups. All other participants who were vaccinated at or before the baseline were excluded from the study. A flow chart outlining the inclusion steps for the final participant samples is available in Supplementary Figures S2, S3. For the CI-DFU, the baseline periods are 10 January to 31 January 2021 for participants aged 65+ years, 1 April to 30 April 2021 for participants aged 50–64 and 1 May to 31 May 2021 for participants aged 18–49. For the CSM, the baseline periods are 25 January to 4 February 2021 for participants aged 65+ years, 29 March to 8 April 2021 for participants aged 50–64 and 3 May to 13 May 2021 for participants aged 18–49.

### Statistical Analysis

In this study, we described participant characteristics and outcomes of interest using counts and percentages, as well as median and interquartile range (IQR). We conducted failure-time analyses for the primary outcomes of interest using Kaplan-Meier curves and log-rank tests. Specifically, we assessed vaccine uptake and SwissCovid app uninstalling outcomes, with the outcomes interchanging as the exposures in two separate substudies. First, we assessed the time to vaccine uptake with the SwissCovid app use as the exposure. All participants were included in this substudy and were separated into app user or app non-user groups based on their reported use of the SwissCovid app over time. Second, we assessed the time to SwissCovid app uninstalling with vaccine uptake as the exposure. Only participants using the SwissCovid app at baseline were included in this substudy and were separated into vaccinated and non-vaccinated participants based on their reported uptake of the vaccine over time. For both outcomes, time of observation started at the date of the inclusion in the study and ended at time of vaccine uptake/app uninstalling or end of follow-up, depending on which occurred first. We assessed precise dates for the vaccine uptake outcome and time-varying app uninstalling from monthly self-reports. In the case of missing data in survey follow-up during the assessment period, we applied the last observation carried forward (LOCF) method to reduce attrition bias (Supplementary Figure S1). For sensitivity analyses, we applied inverse probability of censoring weighting (IPCW) to correct for the possible presence of informative censoring [24].

We further performed multivariable Cox regressions to assess associations between vaccine uptake and app uninstalling with additional variables of interest, stratified by age group and chronic condition to account for varying baseline risks. Specifically, these outcomes were assessed against whether participants reported the following in the past assessment month: (a) frequently or always adhered to preventive measures (i.e., maintaining social distance, only leaving the house when necessary, and avoiding non-essential gatherings with persons outside own household), (b) entered quarantine, (c) got tested for SARS-CoV-2 and (d) received an exposure notification from the SwissCovid app. A detailed description of the choice of the time-varying variables of interest and confounders is in Supplementary Table S1. We reported adjusted hazard ratios (aHRs) with 95% confidence intervals, which represent the weighted average of the true aHRs over the studies’ follow-up periods. We tested the proportional hazard assumption using Schoenfeld residuals. Here, we a-priori expected that the hazards would vary over the follow-up period, as observed in the majority of health-related interventions [25]. For sensitivity analyses, we conducted point-process Poisson regressions to model aHRs over the follow-up period to provide context for possible violations of proportional hazards assumptions from the Cox regressions [26].

All statistical analyses were done in R (version 4.0.0) using ggplot2 (version 3.3.3) for plots and the survival package for survival analyses. Two-sided *p*-values of less than 0.05 were considered statistically significant. Analyses were conducted between January and May 2022.

## Results

### Study Population

A total of 4,514 participants from the CI-DFU and 1,969 participants from the CSM were included in the analysis (flowcharts available in Supplementary Figures S2, S3). In the CI-DFU and CSM cohorts, 64% (2,903/4,514) and 57% (1,125/1,969) of the participants were right-censored, respectively. Demographic baseline characteristics of included participants are reported in Table 1.

**TABLE 1:** Baseline characteristics of participants from the Corona Immunitas Digital Follow Up eCohort study and COVID-19 Social Monitor study studies. Switzerland, January to May 2021.

	CI-DFU (N = 4,514)	CSM (N = 1,969)
Age group		
Ages 18–49	1,170 (25.9%)	914 (46.4%)
Ages 50–64	1,267 (28.1%)	645 (32.8%)
Ages 65+	2,077 (46.0%)	410 (20.8%)
Gender		
Female	2,315 (51.3%)	941 (47.8%)
Male	2,196 (48.6%)	1,028 (52.2%)
Missing	3 (0.7%)	0 (0%)
Language region		
German	4,133 (91.6%)	1,286 (65.3%)
French	381 (8.4%)	391 (19.9%)
Italian	—^a^	292 (14.8%)
Maximum achieved education		
No school certificate	4 (0.1%)	0 (0%)
Compulsory schooling	151 (3.3%)	86 (4.4%)
Secondary degree	2,036 (45.1%)	1,316 (66.8%)
Tertiary degree	2,213 (49.0%)	567 (28.8%)
Missing	110 (2.4%)	0 (0%)
Monthly household income		
<5,000 Fr.	—^b^	452 (23.0%)
<6,000 Fr.	1,389 (30.8%)	—
5,000–9,999 Fr.	—	906 (46.0%)
6,000–9,000 Fr.	1,294 (28.7%)	—
9,000–12,000 Fr.	766 (17.0%)	—
10,000+ Fr.	—	393 (20.0%)
12,000+ Fr.	754 (16.7%)	—
Missing	311 (6.9%)	218 (11.1%)
Current employment status		
Unemployed	2,518 (55.8%)	636 (32.3%)
Employed	1,904 (42.2%)	1,333 (67.7%)
Missing	92 (2.0%)	0 (0%)
Takes ≥ 1 preventive measure against SARS-CoV-2 spread^c^	4,062 (90.0%)	1,793 (91.1%)
SwissCovid app user	2,409 (53.4%)	962 (48.9%)
Has chronic condition^d^	693 (15.4%)	152 (7.7%)

^a^ Responses from Italian language region from CI-DFU were not included due to deviations in content and possible answer choices from the remaining cantons.

^b^ Available answer choices for monthly income differed for CI-DFU and CSM.

^c^ Preventive measures include social distancing, staying home when possible and avoiding non-essential in-person gatherings with persons outside of own household.

^d^ Chronic conditions include cancer, cardiovascular and autoimmune diseases.

Median IQR age in the CI-DFU was 62 (IQR 49–70) years and 51 (IQR 37–61) years in the CSM. For both studies, approximately half of the participants were female (*n* = 2,315, 51% for the CI-DFU and *n* = 941, 48% for the CSM) and most participants resided in the German speaking regions of Switzerland (*n* = 4,133, 92% for the CI-DFU and *n* = 1,268, 65% for the CSM).

For both studies, around half of the participants were self-reported SwissCovid app users at baseline (*n* = 2,409, 53% for the CI-DFU and *n* = 962, 49% for the CSM). Most participants (*n* = 4,062, 90% for the CI-DFU and *n* = 1,793, 91% for the CSM) reported adhering frequently or always to at least one preventive measure against SARS-CoV-2 spread at baseline. Furthermore, 15% (*n* = 693) of the CI-DFU participants and 7.7% (*n* = 152) of the CSM participants reported having at least one severe chronic condition at baseline.

### Description and Failure Time Analysis: Vaccine Uptake

Counts of vaccine uptake outcomes based on SwissCovid app use can be found in Supplementary Table S2. For CI-DFU, the first vaccine was received during the study period by 60% of app users and 58% of app non-users in the 65+ age group, 97% of app users and 85% of app non-users in the 50–64 age group, and 91% of app users and 72% of app non-users in the 18–49 age group. For CSM, the first vaccine was received during the study period by 93% of app users and 87% of app non-users in the 65+ age group, 96% of app users and 76% of app non-users in the 50–64 age group, and 93% of app users and 71% of app non-users in the 18–49 age group.

**FIGURE 2:**
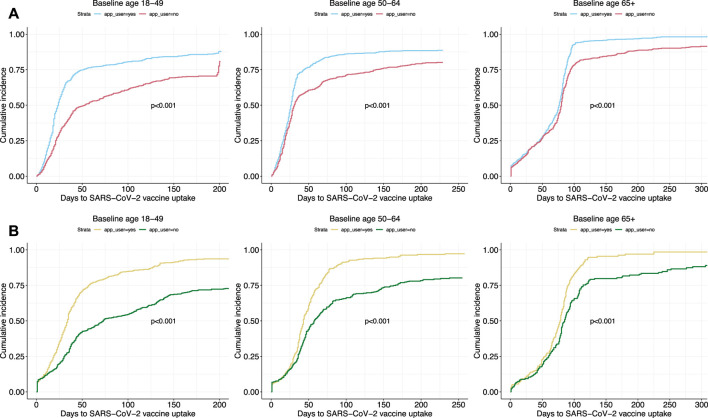
Cumulative hazard curves of vaccine uptake outcomes based on SwissCovid app use. Panel **(A)** curves are from Corona Immunitas Digital Follow Up eCohort study, while Panel **(B)** curves are from the COVID-19 Social Monitor study. *p*-values are retrieved from log-rank tests. Switzerland, January to December 2021.

Kaplan-Meier curves stratified by age groups depicted similar trends in vaccine uptake based on SwissCovid app use in both study cohorts (Figure 2). The cumulative incidence of vaccine uptake in both studies increased rapidly for all age groups in the first days when vaccines were available. A pattern of divergence between app users and app non-users in vaccine uptake was observed. Specifically, vaccine uptake of app non-users was lower over time than app users for all age groups. Sensitivity analyses accounting for informative censoring events through inverse probability of censoring weighting (IPCW) revealed similar results (Supplementary Figure S4).

### Description and Failure Time Analysis: SwissCovid App Uninstalling

Counts of SwissCovid app uninstalling outcomes based on vaccine uptake can be found in Supplementary Table S3. For CI-DFU, the SwissCovid app was uninstalled during the study period by 7.1% of vaccinated and 5.7% of non-vaccinated participants in the 65+ age group, 7.0% of vaccinated and 22% of non-vaccinated participants in the 50–64 age group, and 11% of vaccinated and 28% of non-vaccinated participants in the 18–49 age group. For CSM, the SwissCovid app was uninstalled during the study period by 6.7% of vaccinated and 27% of non-vaccinated participants in the 65+ age group, 5.6% of vaccinated and 46% of non-vaccinated participants in the 50–64 age group, and 13% of vaccinated and 33% of non-vaccinated participants in the 18–49 age group.

**FIGURE 3:**
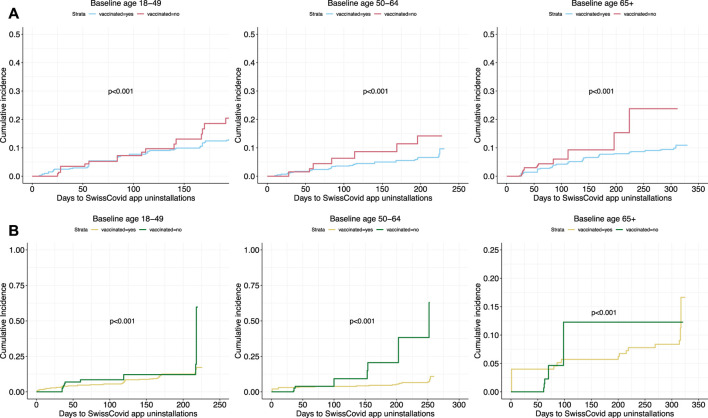
Cumulative hazards curve of SwissCovid app uninstalling based on vaccine uptake. Panel **(A)** curves are from Corona Immunitas Digital Follow Up eCohort study, while Panel **(B)** curves are from the COVID-19 Social Monitor study. *p*-values are retrieved from log-rank tests. Switzerland, January to December 2021.

Kaplan-Meier curves stratified by vaccine uptake depicted similar trends in SwissCovid app uninstalling in both study cohorts (Figure 3). Higher cumulative incidence of app uninstalling was observed over time for participants who did not get vaccinated compared to participants who got vaccinated for all age groups. Sensitivity analyses accounting for informative censoring events through inverse probability of censoring weighting (IPCW) revealed similar results (Supplementary Figure S5).

### Multivariable Analyses: Vaccine Uptake

**FIGURE 4:**
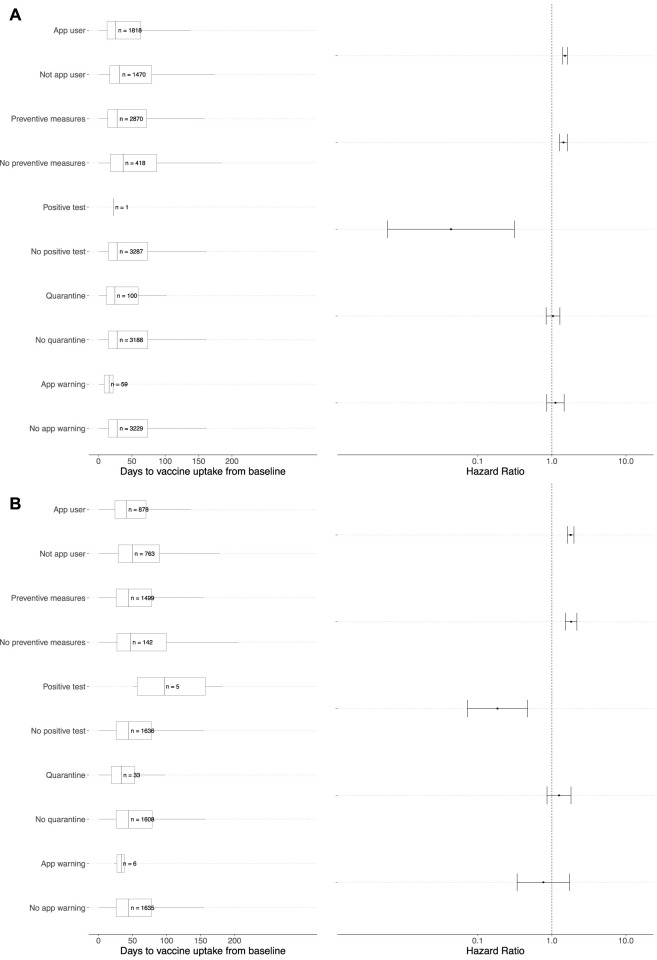
Multivariable Cox proportional hazards regressions and adjusted hazard ratios of vaccine uptake. Panel **(A)** represents the multivariable Cox proportional hazards regression analysis for the Corona Immunitas Digital Follow Up eCohort study, while Panel **(B)** represents the multivariable analysis for the COVID-19 Social Monitor study. Adjusted hazard ratios (aHR) are presented on the right of each panel for event of interest (i.e., vaccine uptake) for the groups with the covariate of interest in comparison to the group without the covariate of interest (e.g., app users vs. nonapp user). For example, in Panel **(A)** the aHR for app users compared to app non-users was 1.51 (95% CI: 1.40–1.62). A detailed description of the choice for each variable of interest in the multivariable Cox regressions can be found in Supplementary Table S1. More information with effect sizes of each variable of interest and reference categories can be found in Supplementary Table S4. Switzerland, January to December 2021.

The multivariable Cox proportional hazards regression model for the vaccine uptake outcome with the time-dependent covariates of interest are displayed in Figure 4. We observed similar findings in both the CI-DFU and CSM studies. In the multivariable analyses, compared to SwissCovid app non-users, app users were more likely to receive their first vaccine dose (CI-DFU: aHR 1.51, 95% CI: 1.40–1.62; CSM: aHR 1.79, 95% CI: 1.62–1.99). Similarly, compared to participants who did not take other preventive measures, participants who frequently or always adhered to other preventive measures were more likely to receive their first vaccine dose (CI-DFU: aHR 1.44, 95% CI: 1.28–1.62; CSM: aHR 1.82, 95% CI: 1.52–2.18). An assessment of the proportional hazards assumption showed that it was fulfilled for the CI-DFU cohort (*p* = 0.25) but was violated for the CSM cohort (*p* = 0.0021). We therefore conducted sensitivity analyses to model the aHRs over the follow-up period through point-process Poisson regressions, which show that the aHRs for vaccine uptake in both cohorts increased over time (Supplementary Figure S6).

### Multivariable Analyses: SwissCovid App Uninstalling Outcome

**FIGURE 5:**
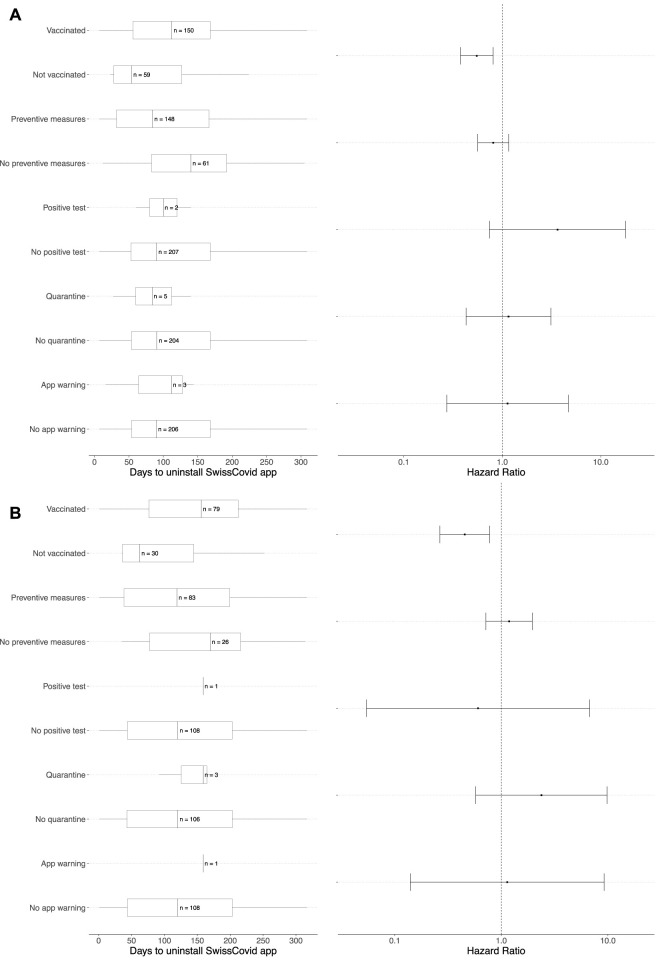
Multivariable Cox proportional hazards regressions and adjusted hazard ratios of SwissCovid app uninstalling. Panel **(A)** represents the multivariable Cox proportional hazards regression analysis for Corona Immunitas Digital Follow Up eCohort study, while Panel **(B)** represents the multivariable Cox proportional hazards regression analysis for COVID-19 Social Monitor study. Adjusted hazard ratios (aHRs) are presented on the right of each panel for the event of interest (i.e., SwissCovid app uninstalling) for the groups with the covariate of interest in comparison to the group without the covariate of interest (e.g., vaccinated vs. not vaccinated). For example, in Panel **(A)** the aHR for participants who received their first vaccine dose in comparison to participants who did not receive their first vaccine dose was 0.55 (95% CI: 0.38–0.81). A detailed description of the choice for each variable of interest in the multivariable Cox regressions can be found in Supplementary Table S1. More information with effect sizes of each variable of interest and reference categories can be found in Supplementary Table S5. Switzerland, January to December 2021.

The multivariable Cox proportional hazards regression model for SwissCovid app uninstalling outcome with the time-dependent covariates of interest are displayed in Figure 5. Compared to participants who did not receive their first SARS-CoV-2 vaccine dose, participants who received their first vaccine dose were less likely to uninstall the SwissCovid app for both the CI-DFU and CSM (aHR 0.55, 95% CI: 0.38–0.81 and aHR 0.45, 95% CI: 0.27–0.78, respectively). An assessment of the proportional hazards assumption showed that it was fulfilled for the CI-DFU cohort (*p* = 0.85) but was violated for the CSM cohort (*p* = 0.0077). We therefore conducted sensitivity analyses to model the aHRs over the follow-up period through point-process Poisson regressions, which show that the aHRs for SwissCovid app uninstalling in both cohorts decreased over time (Supplementary Figure S7).

## Discussion

This study makes use of two independent Swiss nationwide panel studies to assess adherence to preventive measures once SARS-CoV-2 vaccines became available to wider populations in 2021. We observed a dichotomy of participant groups who did not use the digital proximity tracing app during the study period and did not get vaccinated, and another group who used the app during the study period and got vaccinated. Consistent with our hypothesis, we found that participants had a higher chance of using the app or taking additional preventive measures leading up to vaccine uptake. Similarly, participants had a lower risk of uninstalling the app following vaccine uptake.

Our findings reveal that people who use the app and adhere to preventive measures had a higher aHR for vaccine uptake. In particular, we find that individuals who were possibly more concerned about the pandemic, such as those who were older or likely more vulnerable to severe illness, got vaccinated earlier [27]. Our results are aligned with evidence from Caserotti et al. who found that individuals with a high perceived COVID-19 risk score were more likely to get vaccinated and download the digital proximity tracing app in Italy [5]. In the Swiss context, these findings may be indicative of the observed high adherence to preventive measures during the initial phases of the SARS-CoV-2 pandemic in Switzerland [28–30]. They also suggest a possible perceived benefit to combine multiple preventive measures to reduce onward viral transmission leading up to vaccine uptake. This was observed in a study conducted during the first wave of the pandemic in Switzerland, which found that adherence to multiple preventive measures contributed the most to reduce viral transmission, with only 4% of the transmission reduction resulting from natural immunity [31]. This is also aligned with previous studies underscoring the lack of effectiveness of individual preventive measures to reduce onward viral transmission alone [32–35] and the benefits of combining them to enhance each measure [36–39].

We also observed a decreased aHR in SwissCovid app uninstalling after vaccine uptake for both assessed cohorts. This finding suggests a perceived benefit for people to extend their individual protection from vaccines to public-level protection through continued use of the SwissCovid app. In the broader context of the 2021 pandemic in Switzerland, these findings align with the prevailing sentiment at the time that vaccination offered greater personal protection against severe illness rather than population-level protection. In particular, we assessed a period of the pandemic when there were increasing concerns over the vaccine’s ability to reduce the spread of the highly transmissible alpha (widely known as B.1.1.7) and delta (widely known as B.1.617.2) variants [40, 41], and there was only limited evidence on the ability of vaccines to reduce onward viral transmission [42]. At that time, public health guidance in Switzerland kept recommending to adhere to preventive measures despite widespread vaccine rollout, which may explain why individuals were less likely to uninstall the SwissCovid app after getting vaccinated [43].

Contrary to our trade-off hypothesis, we did not find a significant trend of reduced app use after getting vaccinated. However, high counts of app uninstalling were observed, with approximately 7% (pooled study population) of self-reported SwissCovid app users at baseline uninstalling the app following vaccine uptake during the study period. Observed app uninstalling might be due to a lack of perceived benefits from the app or due to experienced difficulties in using the app [8, 44]. App uninstalling among vaccinated participants, particularly, can also be explained by ongoing misconceptions during the alpha and delta variant waves that vaccination could prevent all SARS-CoV-2 infections, making app use seem unnecessary [45]. To address this, future versions of the digital proximity tracing app could incorporate a flexible architecture that integrates additional public health measures. For example, app warnings could be adjusted based on factors such as vaccination status.

Our failure time analyses depicted a dichotomy of participants who did not use the SwissCovid app and did not get vaccinated (16% of pooled study population) versus participants who used the app and got vaccinated (40% of pooled study population) during the study period. The persistent divergence of these two groups over the study period suggests a continued low acceptance of public health measures in reducing viral transmission, such as from the observed negative discourse on the implementation of COVID-19 vaccine certificate in June 2021 [46–49]. On the other hand, we observed that the majority of SwissCovid app users at baseline (approximately 50% of pooled study population) continued using the app and got vaccinated during the study period. When compared to simulations by Ferretti et al., which suggested that at least 70% of the population would need to use the app alone to mitigate viral spread [2], our findings suggest that apps can likely act as complementary measures to vaccines, rather than as standalone measures. In future epidemic contexts, targeted communication strategies can foster trust in using digital proximity tracing apps and highlight their vital role alongside other public health measures, based on their observed interactions [11, 50, 51].

Our study presents some limitations. First, in both the CI-DFU and CSM there may have been self-selection during enrollment that may have led to study participants with higher digital or health literacy, and higher socioeconomic status than the general population. Here, self-selection could have also been in the form of people taking part in the surveys as an additional measure to contribute to the public pandemic response. Second, both panel surveys were based on self-reports and possibly subject to common measurement biases due to socially desirable responding, which may have led to an overestimation of our study’s outcomes of interest. Third, although substantial efforts were presented by the CI-DFU to streamline data collection across various geographical regions in Switzerland, local heterogeneity in the implementation of the questionnaires was not avoidable at times, which may have contributed to disparate results. Fourth, the proportional hazards assumption was violated in the multivariable analyses with the CSM cohorts. However, sensitivity analyses with point-process Poisson regressions suggested the aHR for vaccine uptake increased over time and the aHR for SwissCovid app uninstalling decreased over time, which both confirm the results obtained with the CI-DFU cohort. Fifth, reporting of SwissCovid app uninstalling outcomes may not be suggestive of an active choice to not use the app as a preventive measure anymore but, e.g., participants buying a new phone and not reinstalling the app. Similarly, not reporting SwissCovid app uninstalling outcomes does not necessarily mean that the app was in use. Lastly, our approach to analyze the data with the last observation carried forward (LOCF) method for our exposures of interest may have introduced bias in estimating our study’s effects of interest, even though only a small amount of data in both study cohorts was affected by conditional study participation [52].

### Conclusion

To our knowledge, this study is the first to draw on two nationwide panel studies to assess trends in the use of the digital proximity tracing app in Switzerland, SwissCovid, during the SARS-CoV-2 pandemic in a period when vaccines became widely available. We observed a dichotomy of individuals who did not use the app during the study period and did not get vaccinated, and who used the app during the study period and got vaccinated during the alpha and delta variants of concern. We found a higher probability for vaccine uptake in individuals with app use and adherence to other preventive measures. Furthermore, we found a decreased risk of app uninstalling among individuals with vaccine uptake. Our findings highlight the importance of relevant decision makers to consider possible interactions between different preventive measures in future epidemic contexts. These can inform the development of focused communication campaigns and flexible digital tracing app development that accommodate various preventive measures and their anticipated interactions.
