# Supplementary material for: Interplay of Digital Proximity App Use and SARS-CoV-2 Vaccine Uptake in Switzerland: Analysis of Two Population-Based Cohort Studies
Source: Int J Public Health. 2023 Sep 20;68:1605812. doi: 10.3389/ijph.2023.1605812 (PMC10549773; doi:10.3389/ijph.2023.1605812)
Supplement: Supplementary file 1 [file DataSheet1.docx]

**Supplementary Information**

**Supplementary Figure S1**: Illustration of analyses conducted for censored data

**Supplementary Figure S2**: Participant inclusion for the CI-DFU

**Supplementary Figure S3**: Participant inclusion for the CSM

**Supplementary Table S1**: Subject-knowledge driven variable selection and rationale

**Supplementary Table S2**: Table of exposures and outcomes for vaccine uptake primary outcome

**Supplementary Table S3**: Table of exposures and outcomes for SwissCovid app uninstalling primary outcome

**Supplementary Table S4**: Effect sizes of confounders and variables of interest in exploratory multivariable analyses for vaccine uptake outcome

**Supplementary Table S5**: Effect sizes of confounders and variables of interest in exploratory multivariable analyses for SwissCovid app uninstalling

**Supplementary Figure S4**: Cumulative hazard curves for the Inverse Probability of Censoring Weighting (IPWC) analysis for vaccine uptake outcome based on SwissCovid app use. **Panel A** curves are from the **CI-DFU**, while **panel B** curves are from the **CSM**.

**Supplementary Figure S5:** Cumulative hazard curves for the Inverse Probability of Censoring Weighting (IPWC) analysis for SwissCovid app uninstalling outcome based on vaccine uptake. **Panel A** curves are from the **CI-DFU**, while **panel B** curves are from the **CSM**.

**Supplementary Figure S6**: Point process Poisson regressions for vaccine uptake outcome based on SwissCovid app use. **Panel A** curves are from the **CI-DFU**, while **panel B** curves are from the **CSM**.

**Supplementary Figure S7**: Point process Poisson regressions for SwissCovid app uninstalling outcome based on vaccine uptake. **Panel A** curves are from the **CI-DFU**, while **panel B** curves are from the **CSM**.

This supplementary material has been provided by the authors to give readers additional information about their work.

**Supplementary Figure S1**: Illustration of analyses conducted for censored data


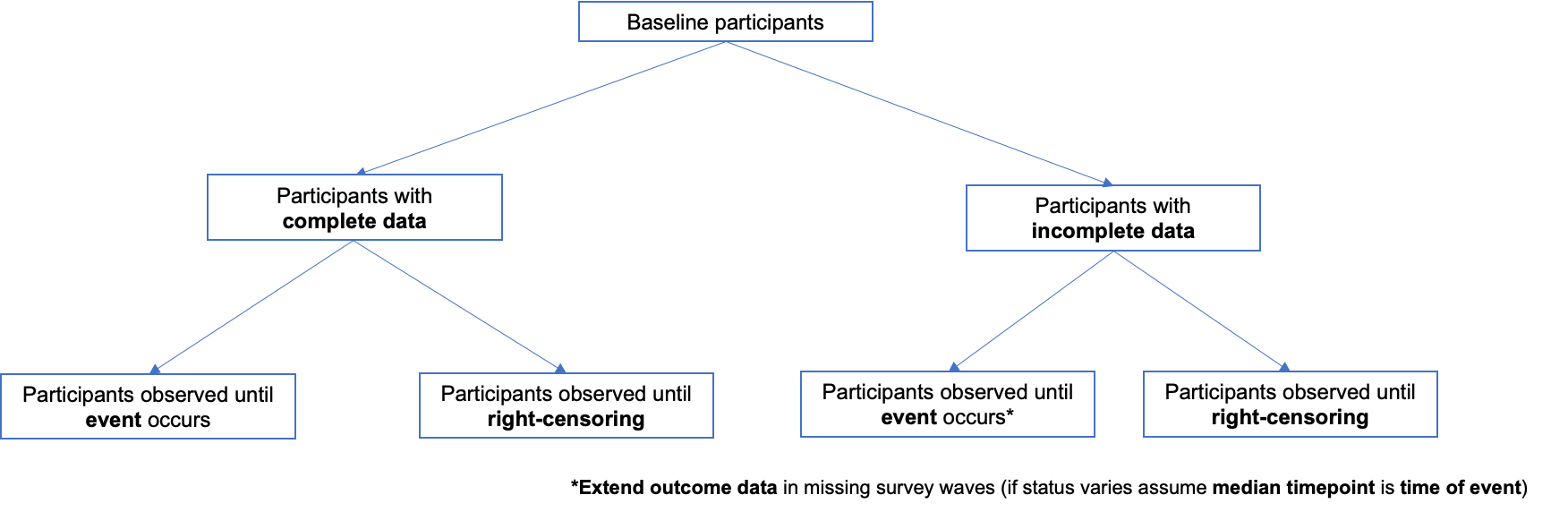


**Supplementary Figure S2**: Participant inclusion for the CI-DFU


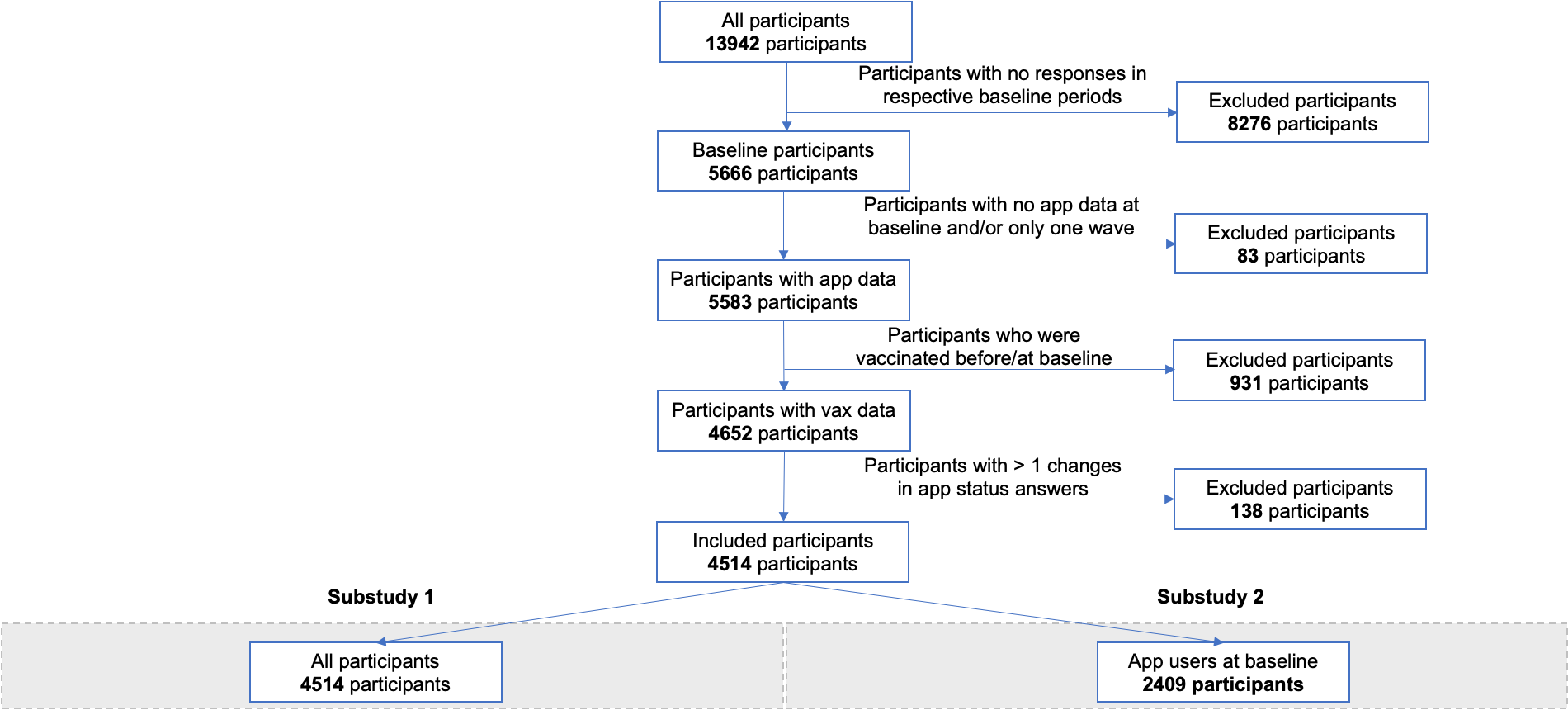


**Supplementary Figure S3**: Participant inclusion for the CSM

**Supplementary Table S1**: Subject-knowledge driven variable selection and rationale

| **Variables** | **Expected Correlation / Comments** |
| --- | --- |
| **Confounders fixed in model:** | |
| Age | The time-varying availability of vaccines based on age group can affect individuals’ choice to keep using digital proximity tracing apps as a preventive measure against SARS-CoV-2. |
| Chronic condition | Higher risk population groups may be more likely to adhere to preventive measures against SARS-CoV-2. |
| Gender | Vaccine hesitancy and uptake of additional preventive measures against SARS-CoV-2 spread has been shown to differ between genders. |
| Education | Individuals with higher levels of education tend to be more compliant with preventive measures against SARS-CoV-2. |
| Employment status | Individuals who work are expected to be healthier, which may have an impact on the extent to which they want to adhere to preventive measures against SARS-CoV-2. |
| Income level | Individuals with higher incomes may have easier means to comply to preventive measures against SARS-CoV-2. |
| Language region | Individuals from different language regions may show differences in acceptance and compliance to preventive measures against SARS-CoV-2. |
| **Exposures / Outcomes of interest** | |
| Vaccine uptake | The use of digital proximity tracing application use may be perceived as a sensible preventive measure both in the absence of vaccine availability or following getting vaccinated against SARS-CoV-2. |
| SwissCovid app uninstalling | The use of a digital proximity tracing application may be perceived as a preventive measure against SARS-CoV-2. |
| **Variables of interest (exploratory analyses):** | |
| Preventive measures | Individuals who adhere to other preventive measures against SARS-COV-2, such as social distancing, are more likely to complement such measures with getting vaccinated or using a digital proximity tracing app on a frequent or consistent basis. |
| Received app warning | Individuals who received a possible exposure to SARS-CoV-2 warning from a digital proximity tracing app may change their adherence to preventive measures, such as by uninstalling the app. |
| Positive test last month | Individuals who tested positive for SARS-CoV-2 may change their adherence to preventive measures. |
| Quarantine last month | Individuals who entered quarantine as a result of a possible exposure to SARS-CoV-2 may change their adherence to preventive measures. |

**Supplementary Table S2**: Table of exposures and outcomes for vaccine uptake primary outcome

***CI-DFU*** *(n=4514)*

|  | SwissCovid app user | Not SwissCovid app user |
| --- | --- | --- |
| Received first vaccine dose | 1734 (38%) | 1413 (31%) |
| Did not receive first vaccine dose | 607 (13%) | 760 (17%) |

***CSM*** *(n=1969)*

|  | SwissCovid app user | Not SwissCovid app user |
| --- | --- | --- |
| Received first vaccine dose | 878 (45%) | 763 (39%) |
| Did not receive first vaccine dose | 66 (3.3%) | 262 (13%) |

**Supplementary Table S3**: Table of exposures and outcomes for SwissCovid app uninstalling primary outcome

***CI-DFU*** *(n=2409)*

|  | Received first vaccine dose | Did not receive first vaccine dose |
| --- | --- | --- |
| Uninstalled SwissCovid | 150 (6.2%) | 59 (2.4%) |
| Kept using SwissCovid | 1662 (69%) | 538 (22%) |

***CSM*** *(n=962)*

|  | Received first vaccine dose | Did not receive first vaccine dose |
| --- | --- | --- |
| Uninstalled SwissCovid | 79 (8.2%) | 30 (3.1%) |
| Kept using SwissCovid | 797 (83%) | 56 (5.8%) |

**Supplementary Table S4**: Effect sizes of confounders and variables of interest in exploratory multivariable analyses for vaccine uptake outcome

| **Variables** | **CI-DFU (n=4154)** | **CSM (n=1969)** |
| --- | --- | --- |
|  | **Multivariable hazard ratio 95% CI** | **Multivariable hazard ratio 95% CI** |
| **Confounders fixed in model:** | | |
| Gender:^a^ male | 1.04 (0.97 - 1.12) | 1.07 (0.97 - 1.18) |
| Gender: not reported | 1.08 (0.15 - 7.73) | n.a. (not available) |
| Education:^b^ mandatory schooling | 0.54 (0.13 - 2.21) | n.a. |
| Education: apprenticeship | 0.57 (0.14 - 2.28) | n.a. |
| Education: matura | 0.63 (0.15 - 2.53) | 1.19 (0.92 - 1.54) |
| Education: higher technical school / university of applied sciences | 0.59 (0.15 - 2.39) | n.a. |
| Education: university | 0.69 (0.17 - 2.76) | 1.46 (1.11 - 1.91) |
| Employment status^c^ | 0.88 (0.80 - 0.98) | 0.92 (0.80 - 1.05) |
| Income:^d^ CHF 3000 - 6000 (CI-DFU) | 1.12 (0.96 - 1.31) | n.a. |
| Income: CHF 6000 - 9000 (CI-DFU)  CHF 5000 - 9999 (CSM) | 1.26 (1.07 - 1.47) | 1.28 (1.12 - 1.46) |
| Income: CHF 9000 - 12000 (CI-DFU)  > 10000 CHF (CSM) | 1.28 (1.08 - 1.51) | 1.74 (1.49 - 2.05) |
| Income: CHF 12000 - 15000 (CI-DFU) | 1.47 (1.22 - 1.77) | n.a. |
| Income: CHF 15000 - 18000 (CI-DFU) | 1.45 (1.17 - 1.79) | n.a. |
| Income: CHF 18000 - 21000 (CI-DFU) | 1.63 (1.21 - 2.19) | n.a. |
| Income: >CHF 21000 (CI-DFU) | 1.65 (1.31 - 2.08) | n.a. |
| Income: not reported | n.a. | 1.17 (0.97 - 1.41) |
| Language region:^e^ German | 0.99 (0.87 - 1.11) | 0.96 (0.84 - 1.11) |
| Language region: French | n.a. | 1.03 (0.87 - 1.22) |
| **Variables of interest (exploratory analyses):** | |  |
| Digital proximity tracing app use | 1.51 (1.40 - 1.62) | 1.79 (1.62 - 1.99) |
| Preventive measures^f^ | 1.44 (1.28 - 1.62) | 1.82 (1.52 - 2.18) |
| Received app warning | 1.12 (0.85 - 1.47) | 0.77 (0.34 - 1.73) |
| Positive test last month | 0.04 (0.01 - 0.31) | 0.18 (0.07 - 0.47) |
| Quarantine last month | 1.04 (0.84 - 1.28) | 1.25 (0.86 - 1.82) |

^a^ Reference: Female gender

^b^ Reference: No school certificate (CI-DFU); Compulsory schooling (CSM)

^c^ Reference: Unemployed

^d^ Reference: < 3000 CHF (CI-DFU); < 5000 CHF (CSM)

^e^ Reference: French region (CI-DFU); Italian region (CSM)

^f^ Reference: Did not take at least one preventive measure

**Supplementary Table S5**: Effect sizes of confounders and variables of interest in exploratory multivariable analyses for SwissCovid app uninstalling

| **Variables** | **CI-DFU (n=2409)** | **CSM (n=962)** |
| --- | --- | --- |
|  | **Multivariable hazard ratio 95% CI** | **Multivariable hazard ratio 95% CI** |
| **Confounders fixed in model:** | | |
| Gender:^a^ male | 0.96 (0.71 - 1.30) | 1.13 (0.76 - 1.68) |
| Gender: not reported | n.a. (not available) | n.a. |
| Education:^b^ mandatory schooling | n.a. (HR < 0.01) | n.a. |
| Education: apprenticeship | n.a. (HR < 0.01) | n.a. |
| Education: matura | n.a. (HR < 0.01) | 1.29 (0.31 - 5.35) |
| Education: higher technical school / university of applied sciences | n.a. (HR < 0.01) | n.a. |
| Education: university | n.a. (HR < 0.01) | 1.16 (0.27 - 4.98) |
| Employment status^c^ | 0.91 (0.59 - 1.42) | 1.02 (0.58 - 1.79) |
| Income:^d^ CHF 3000 - 6000 (CI-DFU) | 1.11 (0.61 - 2.00) | n.a. |
| Income: CHF 6000 - 9000 (CI-DFU)  CHF 5000 - 9999 (CSM) | 0.67 (0.36 - 1.23) | 1.29 (0.72 - 2.31) |
| Income: CHF 9000 - 12000 (CI-DFU)  > 10000 CHF (CSM) | 0.65 (0.34 - 1.23) | 1.67 (0.87 - 3.19) |
| Income: CHF 12000 - 15000 (CI-DFU) | 0.89 (0.44 - 1.79) | n.a. |
| Income: CHF 15000 - 18000 (CI-DFU) | 0.70 (0.30 - 1.61) | n.a. |
| Income: CHF 18000 - 21000 (CI-DFU) | 0.14 (0.02 - 1.12) | n.a. |
| Income: >CHF 21000 (CI-DFU) | 0.66 (0.25 - 1.76) | n.a. d |
| Income: not reported | n.a. | 1.62 (0.78 - 3.34) |
| Language region:^e^ German | 0.98 (0.59 - 1.62) | 1.01 (0.56 - 1.80) |
| Language region: French | n.a. | 0.88 (0.42 - 1.83) |
| **Variables of interest:** |  |  |
| Vaccination status | 0.55 (0.38 - 0.81) | 0.45 (0.27 - 0.78) |
| Preventive measures^f^ | 0.81 (0.56 - 1.16) | 1.18 (0.72 - 1.95) |
| Received app warning | 1.13 (0.27 - 4.68) | 1.14 (0.14 - 9.23) |
| Positive test last month | 3.64 (0.74 - 17.89) | 0.60 (0.05 - 6.73) |
| Quarantine last month | 1.16 (0.43 - 3.12) | 2.38 (0.58 - 9.85) |

^a^ Reference: Female gender

^b^ Reference: No school certificate (CI-DFU); Compulsory schooling (CSM)

^c^ Reference: Unemployed

^d^ Reference: < 3000 CHF (CI-DFU); < 5000 CHF (CSM)

^e^ Reference: French region (CI-DFU); Italian region (CSM)

^f^ Reference: Did not take at least one preventive measure

**Supplementary Figure S4**: Cumulative hazard curves for the Inverse Probability of Censoring Weighting (IPWC) analysis for vaccine uptake outcome based on SwissCovid app use. **Panel A** curves are from the **CI-DFU**, while **panel B** curves are from the **CSM**.

**Supplementary Figure S5:** Cumulative hazard curves for the Inverse Probability of Censoring Weighting (IPWC) analysis for SwissCovid app uninstalling outcome based on vaccine uptake. **Panel A** curves are from the **CI-DFU**, while **panel B** curves are from the **CSM**.

**Supplementary Figure S6**: Point process Poisson regressions for vaccine uptake outcome based on SwissCovid app use. **Panel A** curves are from the **CI-DFU**, while **panel B** curves are from the **CSM**.

**Supplementary Figure S7**: Point process Poisson regressions for SwissCovid app uninstalling outcome based on vaccine uptake. **Panel A** curves are from the **CI-DFU**, while **panel B** curves are from the **CSM**.
